# Does the Current Global Health Agenda Lack Vision?

**DOI:** 10.9745/GHSP-D-22-00091

**Published:** 2023-02-28

**Authors:** Sam L. Forrest, Carmel L. Mercado, Cyril M. Engmann, Andrew W. Stacey, Luxme Hariharan, Sadaf Khan, Michelle T. Cabrera

**Affiliations:** a School of Medicine, University of Washington, Seattle, WA, USA.; b Department of Ophthalmology, University of Washington, Seattle, WA, USA.; c Division of Ophthalmology, Department of Surgery, Seattle Children’s Hospital, Seattle, WA, USA.; d Department of Global Health, University of Washington, Seattle, WA, USA.; e Division of Neonatology, Department of Pediatrics, University of Washington, Seattle, WA, USA.; f PATH, Seattle, WA, USA.; g Division of Pediatric Ophthalmology, Chief of Pediatric Ophthalmology, Nicklaus Children’s Pediatric Specialists, Nicklaus Children’s Hospital, Miami, FL, USA.

## Abstract

Given increasing rates of visual impairment worldwide, we call on national health plans and global development agencies to urgently focus funding and resources toward vision and eye health, with an emphasis on data collection surrounding new and changing burden of eye disease.

## Key Messages

- Despite the successes of the VISION 2020 global initiative, large economic and societal consequences still exist across the life span from vision impairment and blindness, which have not yet been addressed by the global health community.
- There remains a lack of reliable global data on vision health, especially in the case of childhood blindness and vision impairment.
- Historically, vision has been poorly represented in global health budgeting and finance, which objectively reflects its lack of prioritization on the overall global health agenda.
- We need greater investment in cost-effective strategies to prevent and treat a rapidly increasing global burden of ocular disease because of an aging and changing population.

## INTRODUCTION

Visual impairment represents the third leading cause of disability worldwide.^1^ In 2020, an estimated 1 billion individuals lived with preventable or treatable visual impairment globally, with 90% of them living in low- and middle-income countries (LMICs).^2^ Health inequities result in a greater burden of blindness and vision impairment among women and ethnic minorities in all regions of the world.^3^^,^^4^ Visual impairment costs patients, their families, and communities worldwide more than US$3 trillion annually.^5^ Thus, the burden of visual impairment not only affects sight but also hinders the development and progress of entire communities and the broader society.

Because of increased global life expectancy and declining child mortality, global health strategies have expanded to include a “thrive” agenda, addressing chronic disease and disability in addition to survival.^6^ As part of that effort, VISION 2020: The Right to Sight, a World Health Organization (WHO) and International Agency for the Prevention of Blindness global initiative launched in 1999, aimed to eliminate avoidable blindness by 2020.^3^^,^^7^ Although it may have fallen short of its highly ambitious aim statement, VISION 2020 was the first collaborative and interdisciplinary global effort to put vision on the global health agenda, resulting in the successful control, and in many cases eradication, of the leading infectious causes of blindness (trachoma and onchocerciasis).^4^^,^^8^^,^^9^ Furthermore, childhood corneal scarring from Vitamin A deficiency and measles declined as nutritional programs and vaccine efforts increased.^10^ At the time that VISION 2020 was created, an estimated 38 million people were blind and an additional 110 million had moderate to severe visual impairment. Largely because of population growth and changing demographics and without a change in financial investment, these numbers have steadily increased from 1990 to 2020, when an estimated 43.3 million people were blind, with more than 90% having a preventable or treatable cause and an additional 295 million with moderate to severe visual impairment.^3^^,^^11^ Projections estimate that there will be 61 million people blind and 474 million with moderate to severe visual impairment by 2050.^3^^,^^11^ Recognizing the limitations of VISION 2020, the WHO adopted a more focused plan in 2013 at the World Health Assembly, *Universal Eye Health: A Global Action Plan 2014–2019* with a goal to reduce the prevalence of avoidable vision impairment by 25% by 2019 (using 2010 as a baseline).^12^

Data suggest that both the VISION 2020 program and the 2013 WHO–World Health Assembly initiative have not met their goals in large part because the global health community has not kept pace with the dramatic shifts in global burden of disease and growth in an aging population, as well as the lack of financial investment in global vision health. The Global Burden of Disease study found that instead of reaching the WHO target of a 25% global reduction from 2010 to 2019 in avoidable vision impairment, the overall prevalence of avoidable vision impairment increased from 3.92% in 2010 to 4.34% in 2020.^3^ From 1990 to 2020, prevalence of moderate and severe vision impairment in east Asia increased by 10.7%.^3^ Many LMIC national health plans do not include eye care.^4^ From 2014 to 2018, it was estimated that only 0.06% of the total global development assistance was directed to eye health.^4^ These data demonstrate that vision has not been prioritized on the global health agenda despite rapidly increasing global burden of eye disease. Therefore, we must take a closer look at where we have been headed with global vision health and reconsider future directions.

Despite recent data showing the rapidly increasing global burden of eye disease, vision has not been prioritized on the global health agenda.

## CAUSES OF BLINDNESS AND LOW VISION: A CHANGING GLOBAL LANDSCAPE

The causes of blindness and low vision globally have shifted over time because of socioeconomic development and the evolution of disease processes and environmental conditions. Of the estimated 30 million people who were blind in 1990, the main causes included cataract (48.8%), trachoma (15.5%), and glaucoma (13.5%).^11^ Less than 0.1% of cases (360,000) were caused by onchocerciasis (infectious river blindness).^11^ A majority of the cases of childhood blindness (70%) were attributable to vitamin A deficiency.^11^ By 2002, causes of blindness globally included cataract (47.8%), glaucoma (12.3%), and age-related macular degeneration (8.7%). Rates of blindness due to trachoma significantly decreased (3.9%).^13^ Based on limited data, childhood blindness was estimated to affect 1.4 million,^14^ with corneal blindness as a declining cause of blindness in younger age groups from 1993 to 2005, pointing to the success of measles vaccination and vitamin A supplementation projects.^15^

In 2010, the definitions of low vision were revised to include uncorrected refractive error. At that time, the main causes of visual impairment overall were uncorrected refractive error (43%) and cataracts (33%).^16^ The leading causes of avoidable blindness in adults older than 50 years were cataract (51%) and glaucoma (8%), followed by age-related macular degeneration (5%), uncorrected refractive error (3%), trachoma (3%), and diabetic retinopathy (1%).^16^ Among an estimated 18.9 million children with visual impairment in 2010, most were caused by retinopathy of prematurity (ROP), corneal scarring, cataracts, and refractive error/amblyopia.^16^^–^^18^ ROP had entered its “third epidemic.”^19^ Despite rapidly growing rates of preterm infant survival in middle-income countries, only 42% of high-risk infants were estimated to receive appropriate treatment.^20^ As a result, conservative estimates in 2010 suggested that roughly 53,800 preterm infants per year were at risk of lifelong visual impairment from ROP and that ROP was responsible for up to 60% of childhood blindness in middle-income countries.^20^^–^^22^

In addition, urbanization and increased near work led to a rapid increase in the prevalence of myopia and high myopia in schoolchildren, especially in east and southeast Asia, where both genetic and environmental risk factors coexisted.^23^^,^^24^ Global prevalence of myopia increased from 22.9% in 2000 to 28.3% in 2010. Once developed in childhood, high myopia can lead to lifelong increased risk of irreversibly blinding retinal disorders (myopic retinal degeneration and retinal detachment), cataracts, and glaucoma, thereby contributing further to future burden of visual impairment.^17^^,^^23^

By 2020, presbyopia and mild visual impairment were included in epidemiological data for the first time, with an estimated 510 million people affected by uncorrected presbyopia.^3^ Leading causes of blindness were cataract (45%), glaucoma (11%), uncorrected refractive error (6.6%), age-related macular degeneration (5.6%), and diabetic retinopathy (2.5%), with many countries reporting no cases of trachoma or onchocerciasis-related blindness.^25^^,^^26^ Blindness from trachoma decreased by 91% from 2002 to 2020.^4^^,^^8^^,^^9^ Childhood blindness was estimated to affect 1.44 million children, and 22 million had moderate to severe visual impairment.^4^

As shown by these data, disease burden has been shifting from communicable disease and vitamin A deficiency to noncommunicable and age-related conditions. In 2015, an estimated 75% of the global blind population was aged 50 years and older, with associated increasing rates of glaucoma, cataracts, and age-related macular degeneration.^3^ From 1990 to 2015, cataract disability-adjusted life years increased by 89.42%.^27^ The burden of blindness globally is expected to increase exponentially as the population continues to age (the number of people aged 65 years and older is expected to double from 1 to 2 billion over the next 30 years).^3^ Furthermore, as rates of diabetes mellitus continue to increase globally, visual impairment due to diabetic retinopathy is increasing.^28^ From 1990 to 2010, there was already a 64% increase in visual impairment due to diabetic retinopathy. Even when standardized by age, from 1990 to 2020, the world saw a 14.9% increase in visual impairment due to diabetic retinopathy—now the leading cause of preventable blindness in the adult working population.^25^^,^^28^ Finally, with projected increasing urbanization and childhood screen time, a recent meta-analysis predicts that almost half of the world’s population will be myopic by 2050.^24^^,^^29^

## VISION 2020: A CALL-TO-ACTION WITHOUT FUNDING?

In 1999, VISION 2020 initially chose to create a framework for national programs to focus on diseases that cause blindness with proven interventions: cataract, trachoma, onchocerciasis, and vitamin A deficiency. VISION 2020 acknowledged that cataract, glaucoma, and diabetic retinopathy all require an increase in trained ophthalmologists, expansion of clinics and clinical staff, and provision of low-cost surgical services. Rather than including these investments, VISION 2020 placed the burden of funding these programs largely on national resources and patient payment, creating large financial barriers to accessing eye care for LMICs. Studies in sub-Saharan Africa and east Asia have found that cataract surgery, though highly cost-effective on a global or national scale, can impose an out-of-pocket cost as high as half the average annual household income of the patient.^4^

Often, the global health and vision communities work in distinct silos with minimal communication across sectors, centers, and initiatives. Internally, competition for funding between the 2 communities can impede sustainable and large-scale collaborations. Lack of collaboration may be a reason for poor investment in global eye health. Although government expenditure on eye health is not well tracked,^4^ we can extrapolate eye health spending in LMICs based on global health spending data. Such data from 2020 found that donor funding can contribute up to half of total health spending for LMICs; however, external aid has decreased since 2014.^30^ From 2014 to 2018, the average annual funding for all eye health globally was approximately US$102 million—less than 0.06% of total global health funding.^4^ Of this, 66% was spent on elimination of tropical diseases causing blindness,^4^ even though other preventable/treatable forms of blindness were far more prevalent. Based on these data, we conclude that the increasing global burden of eye disease in LMICs is not being adequately addressed by current funding sources. There is an imminent need for more integrated, interdisciplinary, and sustainable initiatives in vision and global health across the life span.

The average annual funding for all eye health globally was less than 0.06% of total global health funding.

## A NEED FOR BETTER DATA

Vision funding, blindness prevention programs, and measuring visual outcomes are critical to meet the growing needs of a rapidly changing global population. Developing and tracking indicators are essential to assessments of whether these programs are achieving their desired effects. Unfortunately, progress indicators established by VISION 2020 were not monitored closely in many countries.^31^ National studies to evaluate vision impairment have been few, of varied methodologies, and lack any pediatric or age-specific focus.^16^^,^^32^ There are limited data from the Caribbean, Central Asia, Latin America, and Central Sub-Saharan Africa, resulting in reliance on nonrepresentative regions to dictate global trends.^3^^,^^32^ The Institute for Health Metrics and Evaluation Financing Global Health database does not include vision health as a separate category in funding.^33^ The top development assistance for health channels’ annual reports fail to mention visual impairment. Where available, visual health data are rarely separated into pediatric and adult populations, despite dramatically disparate diseases, strategies, and implications for these 2 populations.

Uncorrected refractive errors were initially not included in much of the VISION 2020 visual impairment data sets. International studies varied on measuring best spectacle-corrected versus presenting visual acuity.^32^ Presbyopia has remained largely unstudied. The WHO only developed a standardized protocol for data collection in 2015, and near vision impairment was not added to the International Classification of Diseases until 2019.

Addressing childhood vision loss is particularly urgent because of the neuroplasticity of the visual system in children; for example, even uncorrected refractive error can result in permanent vision loss not correctable with glasses due to amblyopia (inadequate central vision development). Nonetheless, low-income countries suffer from limited and unreliable data about childhood blindness and visual impairment.^17^^,^^18^^,^^34^ Rates of childhood blindness are often estimated from data collected from schools for the blind or special education schools; these data are largely considered inadequate due to small numbers and poor representation from the most disenfranchised and rural populations.^15^^,^^17^ Furthermore, cycloplegic eye drops are necessary to accurately assess pediatric refractive error and anisometropia (disparity in refractive error between eyes and a major amblyopia risk factor). For this reason, India created a national consensus to emphasize the correct use of cycloplegia in 2020.^35^ Nonetheless, childhood vision data lack consistent measures of cycloplegic refraction and, therefore, inadequately assesses prevalence of major refractive error or anisometropia.

## RECOMMENDATIONS FOR IMPROVING GLOBAL VISION HEALTH

There are a variety of cost-effective strategies to reduce global visual impairment in the modern era (Table). One is to focus funding on the most rapidly growing and impactful diseases that affect vision. Refractive error is the leading cause of visual impairment and the third leading cause of blindness. Providing adequate spectacle correction for existing uncorrected refractive error is estimated to cost US$28 billion, yet gross domestic product losses from uncorrected refractive error are estimated at US$268.8 billion annually.^36^^,^^37^ Incorporation of photographic eye screening into primary care visits could start to address this issue while also providing for diabetic retinopathy, strabismus, and glaucoma screening in at-risk populations. Access to affordable ophthalmic and optometric care with availability of low-cost spectacles would be essential.

One strategy to reduce global visual impairment is to focus funding on the most rapidly growing and impactful diseases that affect vision.

**TABLE.** Summary of Causes of Avoidable Blindness and Recommendations to Address Them

**Causes of Blindness**	**Recommendations**
**Cataract**	Offer cost-effective cataract surgery programs Provide training that includes teaching cost-effective surgery Include eye health into universal health coverage and national health plans/financing Integrate eye health screening into primary health care services and outreach Expand ophthalmic workforce through increased training and education programs
**Glaucoma**	Provide affordable and accessible preventive screening, monitoring, and treatment options Create vision centers with midlevel personnel for expanded monitoring Conduct research on cost-effective treatments and screening tools
**Uncorrected refractive error**	Offer accurate vision screening in primary care visits and schools Provide low-cost and high-quality spectacles Increase workforce (optometrists, ophthalmic technicians, and ophthalmologists) Ensure private-sector donations and funding
**Age-related macular degeneration**	Offer accessible and affordable preventive screening, monitoring, and treatment options Expand ophthalmic workforce
**Diabetic retinopathy**	Provide low-cost diabetes screening and treatment Offer screening for diabetic retinopathy in primary care settings Build a robust referral network Expand ophthalmic workforce
**Retinopathy of prematurity**	Invest in affordable training and screening programs Offer education on retinopathy of prematurity screening and management Expand ophthalmic workforce
**Corneal scarring**	Mass drug administration programs and distribution of vitamin A where appropriate Public education programs Continue to partner with pharmaceutical industry donations Robust referral network
**Infection/trauma**	Offer mass drug administration programs through community partnerships and pharmaceutical industry donations Train community health workers to identify vision health needs Provide public education and prevention programs and environmental improvement (e.g., SAFE [surgery, antibiotics, facial cleanliness, environment] for trichiasis) Build a robust referral network Collaborate with government-led programs and funders to improve research on prevention and interventions

Cataracts remain the leading cause of blindness and can only be corrected by surgical treatment, requiring the creation of eye care systems and community outreach. In low-income countries, cataract surgery is as cost effective as the bacille Calmette-Guerin vaccine. Cataract surgery in Nepal is the most cost-effective surgical interventions at US$7.29 per disability-adjusted life year averted—equal to cost per disability-adjusted life year averted from bed nets for malaria prevention.^38^

Finally, comprehensive policies and investment in training and screening programs are needed to better address ROP blindness worldwide.^19^^,^^20^ Treatment and screening for ROP in Mexico and the United States were found not only to be cost-efficient but also to create net financial savings when considering the lost productivity of caregivers and the future earning potential of blind individuals.^39^ Investment in well-trained and appropriately distributed ophthalmologists is necessary to adequately care for complex ophthalmic disease in LMICs. Currently, it is estimated that there are only 3.7 ophthalmologists per million people in low-income countries, mostly concentrated in urban areas, while in high-income countries, there are 76.2 ophthalmologists per million people.^40^

Finally, the only way to ensure that progress is being made and that vision health strategies are keeping up with a changing global population is if the global health community actively engages in tracking progress toward specific goals in vision health. Funding and financial interventions related to vision health need to be separately tracked and measured to assess progress. The uncertainties in current data on visual disabilities, particularly among children, could be reduced with population-based studies conducted at the national level following a standardized classification system with results reported by age and sex.

Understanding the rapidly increasing burden of global visual disability as well as projected increases in the future, global financial investment in cost-effective strategies to address vision health is imperative. Furthermore, financial incentives for collaboration across disciplines could break existing silos and create bridges across sectors in vision and global health. The COVID-19 pandemic highlighted global inequities in access to care and the ability to achieve a productive and meaningful life. Vision health across the life span is central to that challenge. We hope that this article serves as a call to action to refocus our global strategy on the rapidly changing landscape of vision health and put sight back on the map.

## Notes

**Peer Reviewed**

**First published online:** January 11, 2023.
